# Transformer-powered precision: A DETR-based approach for robust detection in medical ultrasound with cholelithiasis as a case study

**DOI:** 10.1016/j.csbj.2025.10.037

**Published:** 2025-10-24

**Authors:** Dheeraj Kumar, Mayuri A. Mehta, Heimo Müller, Andreas Holzinger

**Affiliations:** a Department of Information Technology, A. D. Patel Institute of Technology, The Charutar Vidya Mandal University, Anand, India; b Department of Computer Engineering, Sarvajanik College of Engineering and Technology, Surat, India; c Machine Learning and Information Science Group, Diagnostic and Research Institute of Pathology, Medical University Graz, Graz, Austria; d Human-Centered AI Lab, FTEC, Department of Ecosystem Management, Climate and Biodiversity, BOKU University, Vienna, Austria; e Institute of Medical Informatics, Statistics and Documentation, Medical University Graz, Graz, Austria

**Keywords:** Vision Transformer, Detection Transformer, Transfer Learning, Cholelithiasis detection, Ultrasound Image Analysis, Gallstone detection

## Abstract

**Background and objective:**

Transformers have demonstrated strong capabilities in capturing long-range dependencies in visual data, but their application to noisy, low-contrast medical imaging such as ultrasound remains limited. Cholelithiasis detection, often hindered by the inherent limitations of ultrasound imaging, requires more precise and robust computational approaches. This study introduces a detection architecture that integrates convolutional feature extraction with a customized Detection Transformer (DETR) to improve localization and detection in challenging ultrasound conditions.

**Methods:**

The proposed method combines convolutional inductive biases with transformer-based self-attention to capture both local and global spatial relationships. It was applied to the task of detecting gallstones and gallbladder regions in ultrasound images of cholelithiasis. The approach was benchmarked against state-of-the-art object detection models, including RT-DETR, YOLOv8, and YOLO-NAS. Radiologists validated the bounding boxes generated by the model to assess clinical reliability.

**Results:**

The custom DETR achieved confidence score up to 99 % in detecting gallstones and gallbladder regions, outperforming RT-DETR, YOLOv8, and YOLO-NAS. The method demonstrated mean average precision improvements of 13 % and 14 % compared to YOLOv8 and YOLO-NAS, respectively. Radiologist validation confirmed the clinical accuracy and robustness of the proposed detection framework.

**Conclusions:**

By effectively addressing the challenges of low-quality ultrasound imaging, the proposed DETR-based framework provides a reliable and generalizable approach for automated cholelithiasis detection. The findings highlight its potential for integration into real-world diagnostic workflows and its applicability to broader intelligent diagnostic systems at the intersection of computational science, medical informatics, and vision transformers.

## Graphical Abstract

**Figure:**
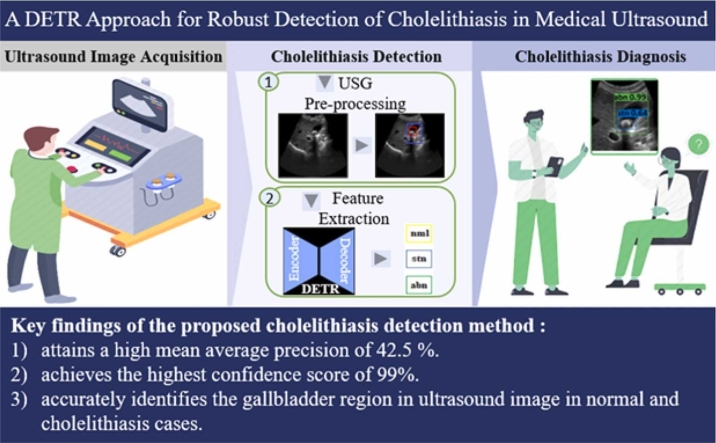

## 1 Introduction

Due to shifting lifestyle patterns globally, the incidence of gallstone disease (cholelithiasis) is steadily increasing [1]. Consequently, precise detection of cholelithiasis and its associated complications is critical for the accurate diagnosis and effective management of gallbladder-related disorders. Given the global health burden posed by these conditions, this topic is of utmost importance, and computational science must play a central role in advancing disease prediction and medical decision-making [2].

Furthermore, the early diagnosis of cholelithiasis is crucial for preventing potential adverse complications such as cholecystitis or pancreatitis [3]. Cholelithiasis refers to stones in the gallbladder or bile ducts. The gallbladder is a small organ that stores bile, a fluid that helps to digest fats [4], [5]. The clinical method to diagnose cholelithiasis involves physical examination, blood tests, and/or gallbladder imaging [4], [6]. Initially, the doctor inquires about the patient's symptoms and family history of cholelithiasis. The symptoms of cholelithiasis include abdominal pain, particularly in the upper right quadrant, nausea, vomiting, indigestion, and rapid weight loss [6]. Furthermore, the doctor performs an abdominal examination to check for abdominal pain and signs of inflammation. In addition, various blood tests are performed to assess liver and bile duct function [7]. However, these findings are not specific to cholelithiasis and may be present in other diseases. In such cases, doctors recommend imaging tests such as Ultrasonography (USG), Computerized Tomography (CT) scan, and Magnetic Resonance Imaging (MRI) [6].

Among various imaging tests, USG is the primary test for gallbladder examination to diagnose cholelithiasis because USG is a non-invasive, widely available, and relatively inexpensive medical imaging test. It is helpful to detect gallstones and assess their size and location [2], [6]. Although ultrasound imaging techniques are beneficial, manual diagnosis of cholelithiasis using USG has several limitations:

1) it is time consuming and expensive [7],
2) it is radiologist-dependent, meaning that the accuracy of the diagnosis can vary depending on the skill of the radiologist [8], [9], [10], and
3) visualizing small gallstones through human-eye is challenging due to low-resolution imaging [3], [5], [9].

Thus, computerization of cholelithiasis detection is required to overcome these limitations of clinical diagnosis.

Computer-aided methods streamline the diagnostic process by allowing timely intervention, reducing the burden on doctors, and improving patient care [11]. Utilizing computer-aided cholelithiasis detection provides valuable decision support to healthcare professionals [12], [13]. Moreover, computer-aided diagnosis is valuable in regions with limited access to specialized radiologists. It also assists healthcare professionals at remote locations in making accurate and appropriate treatment recommendations [11]. Current computerized cholelithiasis detection methods rely on traditional deep learning approaches and conventional image processing techniques, which pose a challenge in handling the inherent complexities and variations in ultrasound images.

With the adoption and advancement in Convolutional Neural Networks (CNNs), cholelithiasis detection using computerized USG analysis has emerged as a promising opportunity for improving diagnostic accuracy and patient care [14], [15]. Utilizing CNN for cholelithiasis detection holds the potential to expedite the diagnosis with higher accuracy. In recent research, pre-trained CNNs, such as Region-based CNN and YOLO, have been mainly used for cholelithiasis detection [16], [17], [18]. However, the need of adequate annotated ultrasound images is a key requirement for fine-tuning pre-trained CNNs effectively [18], [19], [20]. Furthermore, the unavailability of open-source ultrasound image datasets and variations in ultrasound image quality make training CNN challenging [21]. Hence, only a few CNN-based methods have been developed for cholelithiasis detection.

### 1.1 Motivation

The transformer-based architectures, such as Vision Transformer (ViT) [22], DETR [23], Deformable Transformer [24], Data-efﬁcient image Transformer (DeiT) [25], Swin transformer [26], and RT-DETR [27], have demonstrated remarkable success in various computer vision tasks, including disease detection and classification [28], [29], [30]. However, disease detection using these architectures remains challenging due to the inherent limitations of ultrasound imaging, such as low resolution, speckle noise, and structural variability. These factors make it difficult to detect cholelithiasis, especially given the variations in the size, shape, and location of gallstones [16], [18], [21]. Efforts to improve ultrasound image quality through denoising techniques have shown encouraging outcomes. For example, the authors of [31] proposed a denoising method that leverages channel attention and convolutional variational autoencoder to reduce speckle noise while maintaining essential anatomical features. Although ViT and DeiT are optimal solutions for natural image classification, their patch-based processing may miss spatial information crucial for disease detection. Furthermore, swin transformer introduces hierarchical feature maps with a window-based self-attention mechanism to improve over ViT [26], [32]. However, its window-based mechanism generally limits the model’s ability to capture features of tiny objects like gallstones.

DETR, Deformable transformer and RT-DETR introduce an object query mechanism to predict bounding boxes and class labels by bipartite matching. It integrates a CNN backbone to capture low-level spatial features [23], [24], [27]. However, DETR suffers from several limitations which impact its application for cholelithiasis detection: 1) slow convergence, 2) low precision in tiny object detection, and 3) Inefficient in extracting features from low-resolution medical images. The deformable transformer improves the convergence issue of DETR by incorporating multi-scale and deformable attention mechanisms. However, it fails to address challenges specific to ultrasound image analysis. On the other hand RT-DETR utilizes a hybrid encoder and an efficient CNN backbone to achieve real-time performance. However, features extracted from last few layers of CNN backbone may not capture all spatial features and fine-grained details crucial for cholelithiasis detection.

Hence, this paper proposes a custom DETR for cholelithiasis detection from an ultrasound image. The key modifications such as reduced number of encoder layers and object queries made in custom DETR addresses the challenges associated with existing cholelithiasis detection methods and different variants of vision transformer. This could lead to superior cholelithiasis detection compared to traditional approaches.

### 1.2 Contributions

The primary contributions in this paper are summarised below:

1) A novel cholelithiasis detection method using a detection transformer is proposed.
2) The performance of the proposed method is evaluated with two different CNN backbones, namely ResNet-50 and ResNet-101, to identify the most efficient DETR architecture for cholelithiasis detection.
3) The performance of the proposed method is compared with the advanced detection methods namely RT-DETR, YOLOv8, and YOLO-NAS to demonstrate the performance gains.
4) The detection results produced by the proposed cholelithiasis detection method are validated by a team of radiologists to understand their effectiveness in a real-world clinical setting.

### 1.3 Paper structure

The paper is structured in five sections: 2 presents a literature review of the existing work in computer-aided cholelithiasis detection. 3 presents the methodology of the proposed cholelithiasis detection method using DETR. 4 discusses the experimental setup, dataset, results and observation. 5 presents the conclusion and 6 future research directions.

## 2 Background and related work

Deep learning techniques are widely helpful in detecting abnormality in soft organs, such as the gallbladder [3], [8], [9], [12], [15], [16], [17], [18], [33], [34], thyroid gland [35], [36], and lungs [37], using ultrasound images. Most advancements in diagnosing gallbladder diseases focus on tasks such as segmentation [3], [33], classification [3], [15], [16] or abnormality detection [8], [9]. For example, the parameter adaptive pulse coupled neural network has been utilized in [3] to segment gallbladder and gallstone regions. However, this segmentation method exhibits slow processing speed, which limits its real-time applicability in clinical settings. In [33], another gallbladder segmentation method has been proposed to segment the gallbladder in the ultrasound image. In addition to gallbladder segmentation, this method combines principal component analysis with an algorithm to classify gallbladder polyps in neoplastic and non-neoplastic gallbladders. The complexity and variability of gallbladder morphology pose a challenge for this method, resulting in occasional failures in achieving precise segmentation. Furthermore, the integration of principal component analysis with the AdaBoost algorithm introduces additional computational overhead, which leads to increased processing time and resource requirements.

In recent years, transfer learning [38], [39] using pre-trained models has grown faster due to the following advantages offered by them: 1) automatic learning and extraction of relevant features, 2) easy and fast training process due to transfer learning and 3) faster and real-time detection. State-of-the-art work shows that pre-trained CNNs [15], [16], [20], [34] give better detection results for gallbladder diseases than customised CNNs. YOLO-v3 has been utilized in [16] to identify the gallbladder region and classify gallstones from CT image. Similarly, an InceptionV3 model is introduced in [34] to classify gallbladder polyps into neoplastic and non-neoplastic gallbladder from ultrasound image.

Likewise, ResNet-50 is used in [15] to diagnose neoplastic gallbladder polyps and adenocarcinomas from endoscopic ultrasound image. Recently, GBCNet [8] has been proposed to detect Gallbladder Cancer (GBC) from ultrasound image. GBCNet uses a CNN pre-trained on the Common Objects in Context (COCO) dataset [40] to detect the Region of Interest (ROI). Additionally, a curriculum training based on human visual acuity has been proposed to handle spurious textures of ultrasound images. Despite promising benefits from CNN-based detection of gallbladder disease detection, several limitations persist. One notable limitation is the restricted generalizability. Another limitation is the higher computational overhead associated with CNN-based detection methods. The resource-intensive nature of CNN models pre-trained on large datasets like COCO results in extended processing time and increased hardware requirements.

The vision transformer models have shown supremacy in image classification, segmentation and object detection over CNN and pre-trained CNNs. State-of-the-art work shows that transformer-based computer vision models have been primarily used to detect objects in high-resolution natural images. The self-attention mechanisms of transformers have sparked great interest in the medical community to adapt them for capturing long-term dependencies within medical images. Several vision transformer-based methods [9], [41], [42], [43], [44], [45], [46], [47], [48], [49], [50], [51], [52], [53] have been introduced to improve the overall performance in abnormality detection from medical images. Most existing methods combine the transformer with CNN to capture local and global features for better model performance. We critically reviewed these methods, and their comparative analysis is presented in Table 1, Table 2. Table 1 provides the general details of the transformer-based abnormality detection methods. In particular, it includes the method name, year of publication, essential characteristics of the method and the scope of improvement. Table 2 shows a parametric evaluation of the abnormality detection methods listed in Table 1. The parametric evaluation is based on parameters such as the method's purpose, imaging modality, dataset, transformer utilized, and the evaluation metrics used to assess the performance of the transformer model.

**Table 1:** Summary of transformer-based abnormality detection methods.

**Method**	**Year of publication**	**Key characteristics**	**Scope of improvement**
A Distillation Approach to Transformer-Based Medical Image Classification with Limited Data [52]	2025	• Used distillation techniques on DeiT and BERT Pre-Training of Image Transformers (BeiT) to improve classification accuracy. • Used masked image modeling to mask randomly selected patches of the MRI images.	• The model generalizability can be improved by utilizing more MRI images from different demographics. • The distillation techniques can be applied to other transformer-based models.
Enhancing Breast Cancer Detection in Ultrasound Images: An Innovative Approach Using Progressive Fine-Tuning of Vision Transformer Models [53]	2024	• Fine-tuned ViT progressively to detect breast cancer from ultrasound image. • Utilitied dropout and batch normalization layers to improve the model’s generalization capabilities and prevent overfitting.	• The accuracy should be further improved by adding more samples to the dataset. • The progressive fine-tuning process can be extended to other vision transformers.
Transformers with global-local attention for interpretable and accurate gallbladder cancer detection [9]	2023	• Combined the global and local feature maps using a contemporary transformer architecture. • Used a visual bag-of-words style feature embedding the local details to provide explanations of the decision.	• ResNet−101 can be utilized to extract deep global features to improve model specificity. • The RadFormer model can be extended to detect another disease in the gallbladder.
Transformer-based mass detection in digital mammograms [41]	2023	• Used a Shifted Window (Swin) transformer as a backbone to extract features at multiple scales. • Utilized transformer and convolution-based detectors with weighted box fusion to improve the true positive rate.	• The model can be tested on another dataset to demonstrate generalizability on diverse mammogram characteristics. • The performance of the Swin transformer can be compared with different transformer architectures to find the most suitable architecture for mass detection.
Deep learning detection network for peripheral blood leukocytes based on improved detection transformer [42]	2023	• Used pyramid vision transformer as the DETR’s backbone to extract multiscale feature maps. • Used deformable attention module into the DETR to improve the overall model performance. • Employed pre-training on a large general dataset, COCO and fine-tuning on a leukocyte dataset using transfer learning.	• The proposed detection transformer can be directly trained on the leukocyte dataset to understand the model efficacy without pre-training. • Benchmarking against other vision transformer models can provide further insights into the effectiveness of the improved DETR designed for leukocyte detection.
End-to-end transformer for nucleus detection in histopathology [43]	2022	• Proposed NucDETR for Nucleus detection in histopathology images to address challenges like morphological variations and fuzzy boundaries. • Demonstrated the effectiveness of data synthesis for enriching training data and improving model performance.	• NucDETR can be tested on diverse histopathology datasets with different tissue types and disease conditions to assess its robustness and broader applicability.
Lightweight transformer backbone for medical object detection [44]	2022	• Demonstrated significant accuracy gains compared to traditional faster R-CNN and swin transformer model. • Introduced image feature patches in ViT by connecting feature patches of tumors with healthy background of breast images.	• The model's sensitivity to different data augmentation techniques can be explored to optimize detection results.
Vision transformers for classification of breast ultrasound images [45]	2022	• Used pre-trained ViT model to classify breast US images. • Substituted the MLP head with a linear classiﬁer.	• A ViT model can be trained from scratch to improve the accuracy further. • Generative models like GAN can be utilized for image augmentation.
Cross-attention-based multi-scale feature fusion vision transformer [46]	2022	• Combined the strengths of CNN in capturing local details and ViT in capturing long-range dependencies. • Introduced cross-attention between deep and shallow feature maps, enabling effective information exchange to learn discriminative features at different scales.	• Model accuracy can be boosted by modifying the feature extraction process. • Model performance can be evaluated through radiologists in a clinical setting.
Lightweight transformer architecture for detection of COVID−19 [47]	2021	• Replaced the transformer model with a linear transformer model to reduce the space and time complexity of the self-attention procedure from O(n^2^) to O(n). • Presented COVID−19 detection as binary and multiclass classification.	• The linear transformer model can be tested with different transformer architectures. • DETR can be utilized for accurate detection of COVID−19 with bounding boxes.
COVID−19 detection in chest x-ray images using swin-transformer and transformer in transformer [48]	2021	• Used swin transformer and transformer-in-transformer to classify COVID−19, pneumonia and normal images. • Utilized model ensembling using a weighted average to boost the accuracy further.	• More experiments are required to compare the proposed hybrid transformer architecture with other transformer variations. • The model accuracy can be further improved by changing the model hyperparameters.
Transformer for polyp detection [49]	2021	• Used DETR with ResNet−50 for end-to-end polyp detection. • Used • Five-fold cross-validation in the training-validation set to achieve better model generalization.	• ResNet−101 can be utilized as a CNN backbone to compare the detection performance. • Evaluation metrics like precision and recall should be used to evaluate the model.
Lymph node detection in T2 MRI with transformers [50]	2021	• Used DETR with ResNet−50 as CNN backbone for detecting lymph nodes in T2 MRI scans. • Used the bounding box fusion method to reduce the false positive rate. • Used focal loss as bounding box matching cost to overcome the class imbalance problem.	• ResNet−101 can be utilized as CNN backbone to improve the mAP and recall further. • Cross entropy loss can be utilized to compare the performance of focal loss in detection results.
Convolution in Transformer Network (COTR) for end-to-end polyp detection [51]	2021	• Proposed a hybrid framework COTR by embedding convolution layers into transformers. • Utilized convolution layers in the transformer encoders to accelerate the model convergence.	• The concept of embedding convolution layers into encoders can be extended to use in other transformers. • COTR can be tested in other imaging modalities to understand model generalizability.

**Table 2:** Parametric evaluation of transformer-based abnormality detection methods.

**Method [Ref.]**	**Purpose**	**Image modality**	**Dataset**	**Transformer utilized**	**Evaluation metrics**
[52]	Brain Tumor Classification	MRI	Brain Tumor classification dataset	DeiT and BeiT	Accuracy:80.96, Precision:0.86, Recall:0.80, F1 Score: 0.78
[53]	Breast Cancer Detection	Ultrasound	Breast ultrasound images dataset	ViT	Accuracy: 94.49, AUC score: 0.92
[9]	Gallbladder cancer detection	USG	GBCU	RADFORMER	Accuracy:90.2, Specificity:0.90, Sensitivity:0.92
[41]	Mass detection in breast	X-ray	OMI-DB	Swin transformer	True Positive Rate (TPR):78.1, False Positives per Image (FPpI):0.1
[42]	Peripheral blood leukocytes detection from cell	Microscope	Raabin leukocyte	DETR	mAP: 0.961
[43]	Nucleus detection in cell	Microscope	CoNSep, PanNuke	NucDETR	Precision:0.76, Recall:0.86, F1 score:0.81
[44]	Breast cancer detection	X-ray	BCS-DBT	ViT	Average Precision (AP):13.71, AP50:42.04, AP75:4.73, APm:6.20
[45]	Classification of breast cancer	USG	BUSI, Dataset B	ViT	Accuracy:86.7, Area Under the receiver operating characteristic Curve (AUC):0.95
[46]	Classification of breast cancer	USG	ZN-BUSI	ViT	Accuracy:85.33, Precision:0.80, Recall:0.70, F1 score:0.74, AUC:0.92
[47]	COVID−19 detection	USG	POCUS	POCFormer	Accuracy:93.9, Precision:0.90, Recall:0.96, F1 score:0.93, Specificity:0.98, Sensitivity:0.90
[48]	COVID−19 detection	X-ray	Chest XR COVID−19	Swin transformer	Accuracy:94.75, Sensitivity:0.94, Specificity:0.95
[49]	Polyp detection	Colonoscopy	Video frames	DETR	Visual result
[50]	Lymph node detection	MRI	NIH- PACS	DETR	mAP:0.65, Recall:0.91, Sensitivity:0.91
[51]	Polyp detection	Colonoscopy	CVC-ClinicDB, ETIS-LARIB and CVC-ColonDB	COTR	Precision:0.91, Sensitivity:0.93, F1 score:0.92

The literature study shows that the potential usage of DETR in abnormality detection from ultrasound images is relatively underexplored and comparatively limited compared to CNN-based detection methods. Furthermore, DETR presents a frequent issue in detecting tiny objects [51]. DETR may exhibit suboptimal performance when applied to ultrasound images with varying levels of resolution, noise, or artifacts. To overcome the limitations of existing DETR models, this paper aims to design an efficient DETR model for cholelithiasis detection. Unlike the conventional object detection methods, the detection transformer incorporates an encoder to model the relationship between image features extracted by a CNN backbone, a decoder to generate object queries, and a feedforward network to assign class labels and bounding boxes around the detected objects. It is incredibly effective, as it allows more efficient and accurate detection of objects in ultrasound image. This paper presents the first effort to examine the application of detection transformers for cholelithiasis detection from ultrasound image.

## 3 The proposed cholelithiasis detection using DETR

**Fig. 1:**
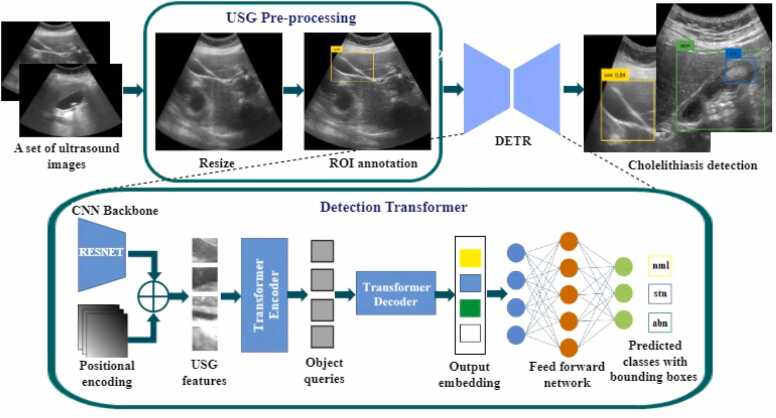
The flow of proposed cholelithiasis detection using DETR from ultrasound image.

A novel cholelithiasis detection method using DETR is proposed in this section. Fig. 1 shows the flow of the proposed cholelithiasis detection using DETR from an ultrasound image. Table 3 represents the notations used in this paper. The proposed method comprises two significant steps: 1) ultrasound image pre-processing and 2) cholelithiasis detection using a detection transformer. Algorithm 1 outlines the pseudocode of the steps given in Fig. 1.

Algorithm 1 Proposed cholelithiasis detection using DETR from ultrasound image

**Figure:**
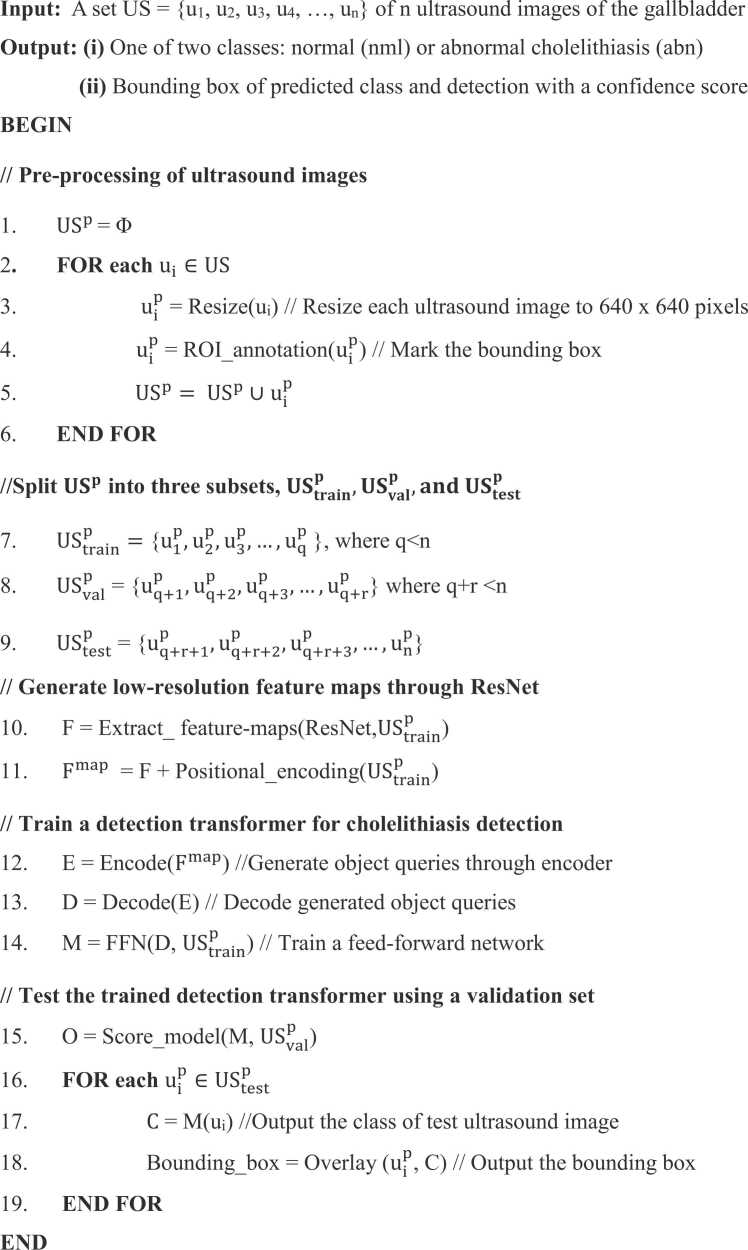

**Table 3:** The list of notations.

**Notation**	**Description**
n	Total number of ultrasound images
US = {u_1_, u_2_, u_3_, …, u_n_}	A set of ultrasound images of the gallbladder
${\mathrm{US}}^{p}\;$= {$u_{1}^{p},u_{2}^{p},u_{3}^{p},\;\ldots ,\;u_{n}^{p}$}	A set of pre-processed ultrasound images
${\mathrm{US}}_{\mathrm{train}}^{p}$	A subset of pre-processed ultrasound images for training
${\mathrm{US}}_{\mathrm{val}}^{p}$	A subset of pre-processed ultrasound images for validation
${\mathrm{US}}_{\mathrm{test}}^{p,}$	A subset of pre-processed ultrasound images for testing
q	Total number of ultrasound images for training
r	Total number of ultrasound images for validation
F = {$f_{1}$, $f_{2}$,$\;f_{3}\;,\;\ldots ,\;f_{k}$}	A set of k feature maps generated through ResNet
$F^{\mathrm{map}}\;$= {$f_{1}^{\mathrm{map}},f_{2}^{\mathrm{map}},\;\ldots ,\;f_{k}^{\mathrm{map}}$}	A set of k feature maps with positional encoding
E	Object queries generated through the encoder
D	Output embedding generated through the decoder
M	Detection transformer model
O	Prediction value
nml	Normal gallbladder
abn	Abnormal gallbladder with cholelithiasis
stn	Gallstone

**Fig. 2:**
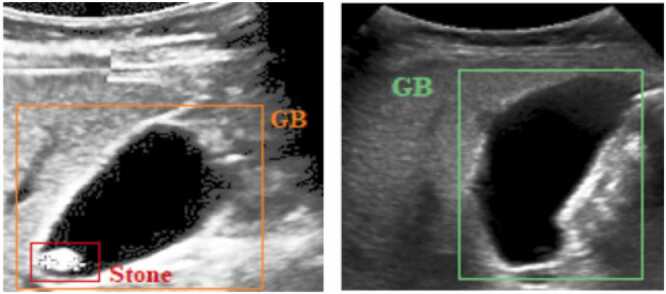
ROI annotation of the gallbladder.

The input to Algorithm 1 is a set US = {u_1_, u_2_, u_3_, …, u_n_} of n ultrasound images. Steps 1–6 of Algorithm 1 represent the pre-processing of ultrasound images. These steps are crucial for improving the quality of ultrasound images for further analysis and interpretation. The pre-processing starts with image resizing, followed by the annotation of the ROI with the class label assignment. Each image is resized to make all images uniform in height and width. All images are resized to 640 × 640 pixels for a uniﬁed image size and converted into COCO format. After resizing, class labels, normal (nml) and abnormal cholelithiasis (abn), are assigned to each ultrasound image. Additionally, ROI is annotated using a bounding box that spans the gallbladder and gallstones. The bounding box of gallstones is represented using the label ‘stn’ for the abnormal cholelithiasis class. The bounding-box annotations of ROI consist of four coordinates aligned with the X and Y axes. Step 5 of Algorithm 1 shows that pre-processed ultrasound images are stored in the set US^P^. Fig. 2 demonstrates samples of annotated ultrasound images for each class.

Next, the images from the pre-processed set ${\mathrm{US}}^{p}$ are divided into three subsets, ${\mathrm{US}}_{\mathrm{train}}^{p},\;{\mathrm{US}}_{\mathrm{val}}^{p},\;{\mathrm{and US}}_{\mathrm{test}}^{p,}$ for training, validation, and testing, respectively, as given in steps 7–9 of Algorithm 1. Once the ROI annotation is done for each input ultrasound image, a detection transformer is trained to identify the region of gallbladder and gallstone with their respective classes and detect cholelithiasis from the ultrasound image. Detection transformer is an object detection method that uses a transformer-based architecture to perform end-to-end object detection. The proposed detection transformer for cholelithiasis detection consists of four components: 1) a CNN backbone, 2) an encoder, 3) a decoder and 4) a feed-forward network.

The CNN backbone is responsible for learning the features present in ultrasound images. As shown in Steps 10 and 11 of Algorithm 1, a pre-trained CNN of ResNet family has been used as a CNN backbone, which outputs a low-resolution feature map F. ResNet models, particularly with their residual connections, are well-known for effectively extracting features even from smaller datasets. These residual connections allow the network to learn more complex representations of gallstone and gallbladder. Additionally, ResNet helps in retaining deep representation of complex textures and spatial information about the gallbladder. The set of feature maps serves as the visual representation of the ultrasound image. The feature map extracted from CNN is flattened and transposed to obtain a one-dimensional representation of feature maps. Since transformers lack positional information, positional encodings are combined with the feature maps to ensure the model can distinguish between different object locations in the image. Next, the sequence of features with positional encoding is processed by the transformer encoder that generates a set of object queries E.

The transformer encoder consists of multiple self-attention layers and feed-forward neural networks, allowing the model to capture global and contextual information. The object queries generated earlier are passed through a decoder for bipartite matching of the most relevant positions in the image with each object query. This matching is achieved through a self-attention mechanism of the decoder. Furthermore, a feed-forward network is trained to classify each object query into one of the classes and predict the bounding boxes for each query. The class predictions represent the probability distribution over the given classes of cholelithiasis, while the bounding box predictions represent the coordinates of the predicted bounding boxes of gallbladder and gallstone, if any. Finally, the DETR model returns a bounding box with a predicted class and confidence score for the detected region of the gallbladder in the ultrasound image. The confidence score is the probability of the model correctly detecting the object.

The model is trained through bipartite matching loss, computed between the object queries and the ground-truth bounding boxes. The matching process assigns each object query to the most relevant object in the image. The number of queries is set to three, which determines the maximum number of objects to be detected in a single image. Furthermore, the Intersection over Union (IoU) metric is used to find the similarity of the predicted bounding box $\bar{b},\;$among the object queries for each ground-truth bounding box b. The similarity metric is calculated using the overlap between two bounding boxes for each image from the training set ${\mathrm{US}}_{\mathrm{train}}^{p}$. Then, the Hungarian matching algorithm finds an optimal one-to-one mapping of each query to the given object. Once all pairs are matched, the next step is to compute the Hungarian loss, given in Eq. 1. Hungarian loss is computed as a linear combination of a negative log-likelihood for class prediction and bounding box loss. The bounding box loss defined in Eq. 2 is a linear combination of the intersection over union loss and L1 regularisation. The bounding box loss optimizes the detected bounding boxes. The cross-entropy loss defined in Eq. 3 is also used to optimize model parameters for class prediction of detected objects.

(1) $$L_{\mathrm{Hungarian}}=\sum_{i=1}^{N}\left\{-\log\left({p(y}_{i}\right)\right)+L_{\mathrm{boundingbox}}(b_{i},\bar{b_{i}}\;)\}$$

Where p(y) is the predicted probability of a given class for all N inputs.$\;L_{\mathrm{boundingbox}}$ denotes the bounding box loss computed using predicted bounding box $\bar{b}$, and ground-truth bounding box b.

(2) $$L_{\mathrm{boundingbox}}(b_{i},\bar{b_{i}}\;)=\mathrm{IoU}(b_{i},\bar{b_{i}})+∆L_{1}\left\|b_{i}-\bar{b_{i}}\right\|$$

Where $\mathrm{IoU}\left(b_{i},\bar{b_{i}}\right)=\frac{\mathrm{Area of intersection}(b_{i},\bar{b_{i}})}{\mathrm{Area of union}(b_{i},\bar{b_{i}})}$ and $∆L_{1}$denotes L1 regularisation loss. Area of intersection$\left(b_{i},\bar{b_{i}}\right)$ is the area where the bounding boxes $b_{i}$ and $\bar{b_{i}}$ ovelap. Similarly, Area of union$\left(b_{i},\bar{b_{i}}\right)$ is the total area covered by both bounding boxes $b_{i}$ and $\bar{b_{i}}$.

(3) $$L_{\mathrm{crossentropy}}=-\frac{1}{N}\sum_{i=1}^{N}y_{i}.\log({p(y}_{i}))+(1-y_{i}).\log\left(1-{p(y}_{i})\right)\;$$

Where y is the class label and p(y) is the predicted probability of a given class for all N inputs.

## 4 Implementation and analysis

4.1 presents the experimental setting and the systematic flow of experiments. 4.2 presents the details of the dataset used in each experiment. 4.3 outlines the evaluation metrics used to investigate the performance of the proposed method. 4.4 discusses the obtained results.

### 4.1 Experimental setup

We use PyTorch version 2.0.5 as a Python platform, and GPU supported machine with NVIDIA Cuda 11.8 compiler for all four experiments. Table 4 presents the hyperparameters, and their values. The first experiment aims to detect cholelithiasis using DETR with ResNet-50 as the CNN backbone, while ResNet-101 is used as the CNN backbone in the second experiment. CNN backbone is used for extracting low-resolution feature maps from ultrasound images. The learning rate of ResNet is initialized to 10^−5^. The encoder and decoder of proposed DETR are trained using the AdamW optimizer, with an initial learning rate of 10^−4^. The batch size is set to 4 with 50 epochs of training. Finally, the class prediction is computed by a three-layer feed-forward network with a ReLU activation function.

**Table 4:** Hyperparameters with tuned values.

**Experiment no.**	**Model**	**Optimizer**	**Learning rate**	**Epochs**	**Batch size**
**1**	DETR with ResNet−50	AdamW	10^−4^	50	4
**2**	DETR with ResNet−101				
**3**	RT-DETR		10^−3^		
**4**	YOLO-v8	Adam	10^−5^		16
**5**	YOLO-NAS				

RT-DETR [27] is used as a benchmark to demonstrate the effectiveness of the proposed DETR-based method. RT-DETR utilizes deformable attention for faster convergence and real-time inference. Third experiment aim to implement RT-DETR and compare it’s performance with the proposed method. Recently, YOLO models [19] received significant attention from the research community for object detection due to their precise and accurate detection results. Hence, in our fourth experiment, we use YOLO-v8, trained on the COCO [40] dataset, to detect cholelithiasis. YOLO-v8 was released in January 2023 by Ultralytics. YOLO-v8 combines high-level features with contextual information to improve the detection accuracy of objects. Similarly, the fifth experiment implements YOLO-Neural Architecture Search (YOLO-NAS) using transfer learning. YOLO-NAS was released in May 2023 to improve the localization of small objects and enhance the detection performance for real-time edge-device applications. The batch size is 16, with 50 training epochs for both YOLO models. We used the same data split for all experiments described in 4.2.

### 4.2 Dataset statistics

**Fig. 3:**
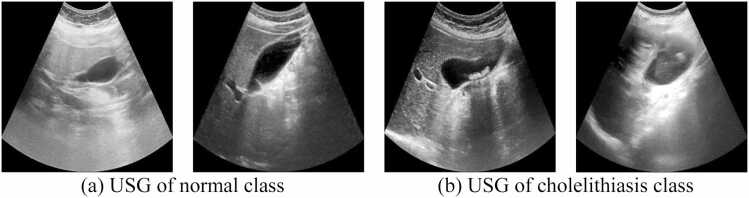
Sample ultrasound images used in experiments.

The statistics of the dataset are represented in Table 5. The dataset consists of 900 abdominal ultrasound images of 218 patients from the Post-graduate Institute of Medical Education and Research (PGIMER) located in Chandigarh city of Punjab state of India, 273 ultrasound images of 30 patients from SIDS Hospital and Research Centre located in Surat city of Gujarat state of India and 37 ultrasound images of 15 patients from Parul Sevashram Hospital located in Vadodara city of Gujarat state of India. Fig. 3 shows sample ultrasound images from the dataset. A total of 598 ultrasound images of normal gallbladder and 612 ultrasound images of cholelithiasis (gallbladder with gallstone) are collected. Out of 1210 images, 787 (65 %) ultrasound images are utilized for training, 302 (25 %) images for validation, and the remaining 121 (10 %) images are used for testing to measure the model's performance. The distribution of ultrasound images of the normal class and cholelithiasis class is presented in Table 6. As shown in Table 6, 1210 ultrasound images are distributed in the training, validation, and test sets.

**Table 5:** Dataset description.

	**PGIMER, Chandigarh**	**SIDS Hospital & Research Centre, Surat**	**Parul Sevashram Hospital, Vadodara**	**Total**
Number of patients	218	30	15	263
Number of ultrasound images of normal gallbladder	402	194	2	598
Number of ultrasound images of abnormal gallbladder with cholelithiasis	498	79	35	612
**Total number of ultrasound images**	900	273	37	**1210**

**Table 6:** Distribution of ultrasound images in training, validation and test sets.

	**Normal class**	**Cholelithiasis class**	**Total**
**Training set**	422	365	787
**Validation set**	116	186	302
**Test set**	60	61	121
**Total**	598	612	1210

### 4.3 Evaluation metrics

The performance of the proposed cholelithiasis detection method is assessed by utilizing three widely used evaluation metrics: precision, recall, and Mean Average Precision (mAP). As defined in Eq. 4, precision is the ratio of truly predicted positive ultrasound images, and the predicted positive ultrasound images in each class. Precision is used to accurately describe the purity of truly positive detections relative to the ground-truth USG. Similarly, recall, defined in Eq. 5, also known as sensitivity, is the ratio of correctly predicted positive ultrasound images divided by the total number of images. The mAP is calculated using an average of the Average Precision (AP) metric across each class, as given in Eq. 6.

(4) $$\mathrm{Precision}=\frac{\mathrm{TruePositive}}{\mathrm{TruePositive}+\mathrm{FalsePositive}}$$

(5) $$\mathrm{Recall}=\frac{\mathrm{TruePositive}}{\mathrm{TruePositive}+\mathrm{FalseNegative}}$$

(6) $$\mathrm{mAP}=\frac{1\;}{N}\sum_{i=1}^{N}{\mathrm{AP}}_{i}$$

### 4.4 Results and discussion

**Fig. 4:**
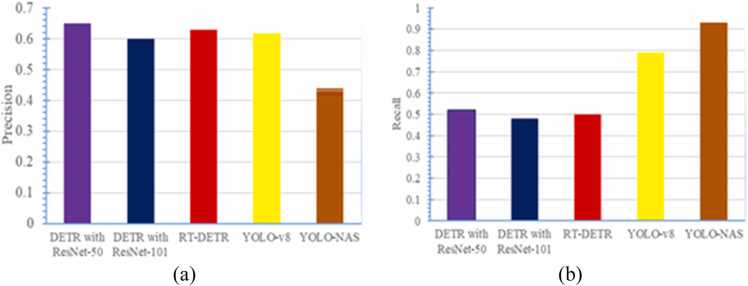
Comparative analysis of precision and recall of proposed cholelithiasis detection using DETR and YOLO models: (a) Precision plot and (b) Recall plot.

**Fig. 5:**
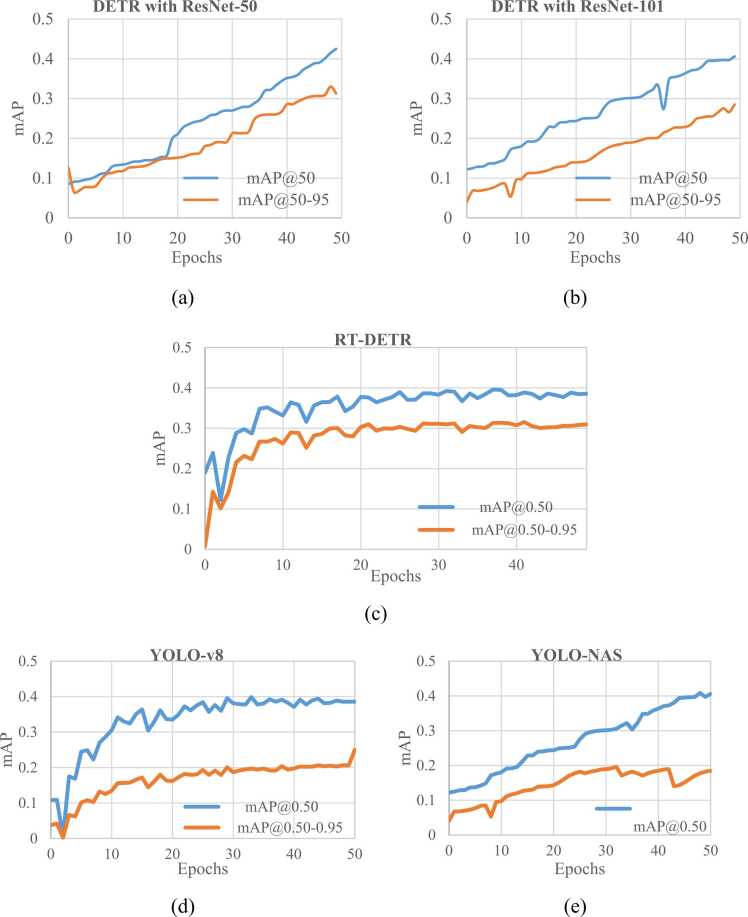
Comparative analysis of mAP results with confidence 0.50 and 0.50–0.95: (a) mAP plot of DETR with ResNet-50, (b) mAP plot of DETR with ResNet-101, (c) mAP plot of RT-DETR, (d) mAP plot of YOLO-v8, and (e) mAP plot of YOLO-NAS.

The results of the five experiments described in 4 are reported in Table 7. Additionally, Table 7 presents the comparative analysis of the proposed method using DETR and YOLO models for cholelithiasis detection from ultrasound images. Figs. 4(a) and 4(b) show a comparative analysis of precision and recall attained in each cholelithiasis detection model. Further, Fig. 5 illustrates a comparative analysis of mAP with respect to the epochs of each experiment. The x-axis represents the epochs during training, and the y-axis denotes the mAP. Figs. 5(a) and 5(b) show that the proposed method using DETR with ResNet-50 exhibits a higher mAP than DETR with ResNet-101. Fig. 5(c) shows the plot of mAP of RT-DETR. Despite being slower, the proposed method outperforms RT-DETR in cholelithiasis detection. Figs. 5(d) and 5(e) show that YOLO-v8 exhibits higher mAP with minor variation than YOLO-NAS. The results show that the proposed method performs significantly better than the YOLO models.

**Table 7:** Results of proposed cholelithiasis detection using DETR and YOLO models.

**Model**	**Trainable parameters**	**Model size**	**Precision**	**Recall**	**mAP**	
					**@0.50**	**@0.50–0.95**
DETR with ResNet−50	41.3 M	166.01 MB	0.65	0.52	0.43	0.32
DETR with ResNet−101	60.2 M	241.76 MB	0.60	0.48	0.41	0.27
RT-DETR	32.81 M	188 MB	0.63	0.50	0.40	0.30
YOLO-v8	43.6 M	174.4 MB	0.61	0.79	0.38	0.24
YOLO-NAS	66.91 M	256 MB	0.42	0.91	0.40	0.18

Surprisingly, YOLO-NAS attains a recall of 0.91, the highest among the other models. Relatively low recall performance of DETR models raises clinical concerns. The performance difference likely stems from DETR’s global attention mechanism, which effectively reduces false positives. However, it is less capable of detecting small, low-contrast features from ultrasound images with natural variations. The recall of DETR-based models can be improved by extending training epochs, integrating attention mechanisms designed for small-object detection, and using relatively larger datasets. These efforts will help balance sensitivity, precision, and model efficiency for real-world deployment in diverse clinical settings.

Although the YOLO models achieve higher recall values, the trainable parameters and model size increased significantly compared to the proposed DETR models. The size of the model highly increases the time required for training and the overall cost of deployment. Moreover, the increased model size impacts the inference speed and memory footprint, posing challenges for deployment in resource-constrained environments such as portable ultrasound devices and real-time clinical settings. In contrast, the proposed DETR-based approach strikes a balance by maintaining a relatively compact architecture, enabling faster training and more efficient deployment without sacrificing precision, although further work is needed to improve its recall.

Moreover, transformers typically require large amounts of data to perform well, but our dataset contains only 1210 ultrasound images from 263 patients. Although clinically validated, the limited size of dataset may increase the risk of overfitting for transformer-based models. Moreover, the dataset was collected from a specific geographic region and clinical environment, which may limit the DETR model’s ability to perform well on ultrasound images from other clinical settings. To mitigate these risks, future work will focus on expanding the dataset to include patient demographics, and ultrasound machine settings across different clinical environments. Expanding the dataset and incorporating cross-domain training strategies would further enhance the model’s clinical reliability.

**Fig. 6:**
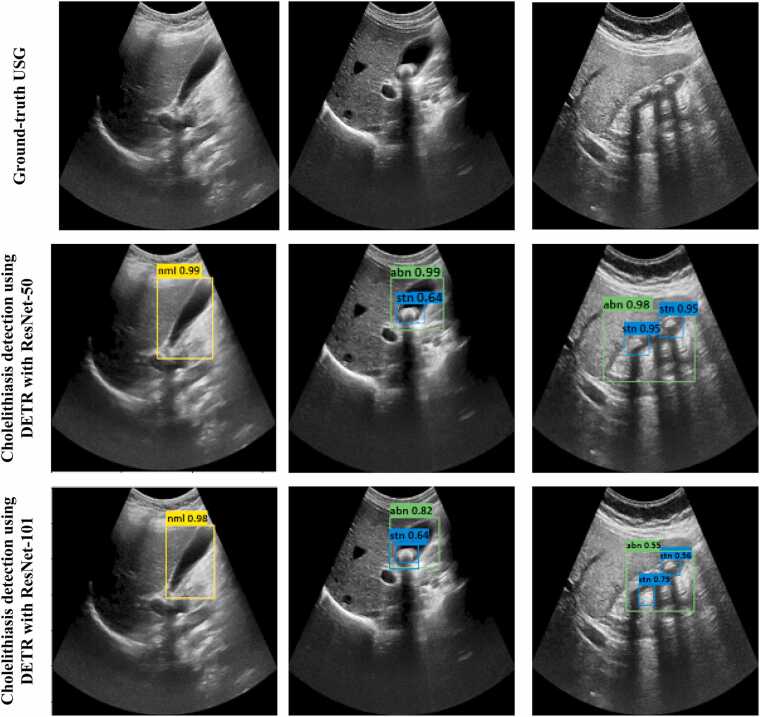
Visual results of proposed cholelithiasis detection method using DETR with ResNet-50 and ResNet-101.

**Fig. 7:**
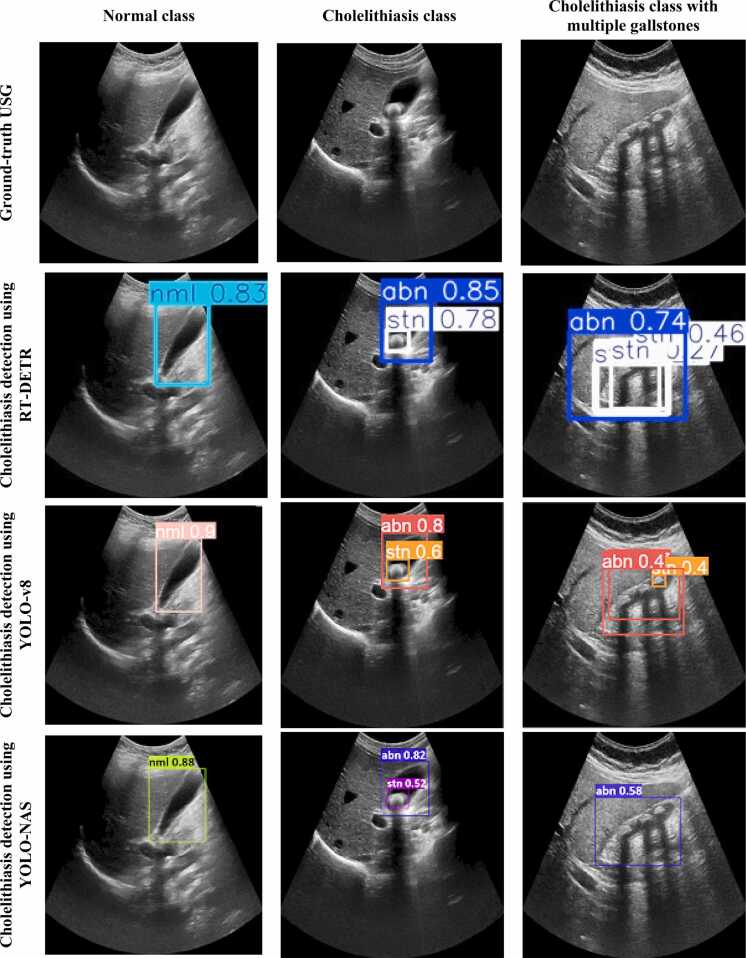
Visual results of cholelithiasis detection using RT-DETR, YOLO-v8 and YOLO-NAS.

**Fig. 8:**
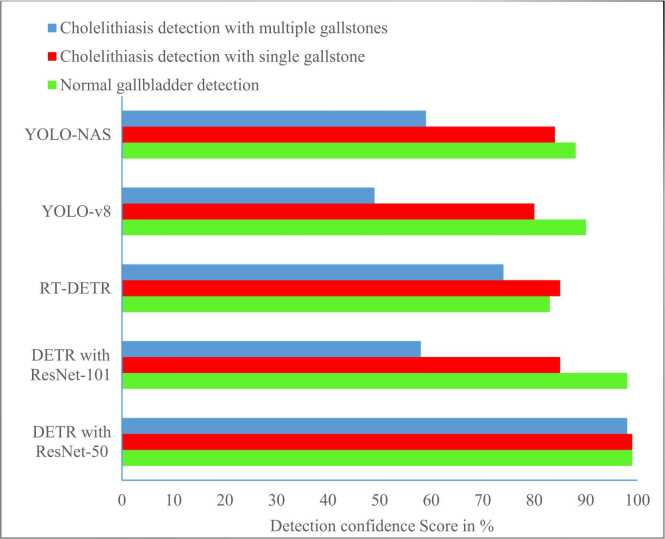
Comparative analysis of detection confidence score for each class of ultrasound images.

Fig. 6 and Fig. 7 demonstrate bounding box predictions of cholelithiasis detection with conﬁdence score. Fig. 8 shows a plot of the detection conﬁdence score in each class of ultrasound images. The proposed cholelithiasis detection using DETR with ResNet-50 achieved the best performance with the highest confidence score of 0.99 for normal and cholelithiasis detection with a single gallstone and 0.98 for cholelithiasis detection with multiple gallstones. While the proposed DETR with ResNet-50 demonstrates a high confidence score for individual cholelithiasis detection, the overall detection performance as measured by mAP remains limited. This highlights the difference between model confidence in single predictions and the aggregate performance that accounts for both precision and recall across the dataset. Additionally, the current evaluation is limited to a relatively small dataset. The model’s performance on more diverse populations, different ultrasound machines, and varied imaging protocols remains to be validated.

The experimental results show that the low-level visual features are better reﬂected in ResNet-50. Hence, the detection accuracy of the proposed method is better when ResNet-50 is used with DETR. Although ResNet-50 captures low-level visual features effectively, there is potential to further enhance feature representation by exploring more advanced backbone architectures. To ensure clinical reliability, radiologist validation was carried out in collaboration with three independent clinical sites in India: PGIMER Chandigarh, SIDS Hospital and Research Centre Surat, and Parul Sevashram Hospital Vadodara. A total of three experienced radiologists participated in the study. Their experience over 10 years in abdominal ultrasonography. Each radiologist independently annotated gallbladder and gallstone regions on 121 ultrasound images used for testing. The annotations were compared with the model-generated bounding boxes to assess concordance and clinical plausibility.

Inter-rater agreement among the radiologists was evaluated using Cohen’s kappa (κ) for pairwise comparisons. The κ statistic was interpreted following the conventional scale: κ < 0.4 (poor), 0.41–0.6 (moderate), 0.61–0.8 (substantial), and > 0.8 (almost perfect agreement). The observed κ value of 0.78 indicated substantial agreement, confirming the consistency of expert annotations. Furthermore, the agreement between the model predictions and the consensus annotation (majority vote of radiologists) is quantified using the IoU metric that demonstrate close alignment with clinical assessments.

## 5 Conclusion

Accurate identification of cholelithiasis from ultrasound images is essential because cholelithiasis is a severe disease with a high mortality rate. Cholelithiasis often poses challenges in diagnosis, particularly in cases of small or obscured stones. In this paper, we have presented a novel method of cholelithiasis detection using a detection transformer. Through extensive experimentation and evaluation, we have shown that detection transformers surpass in capturing complex spatial dependencies and patterns from ultrasound image, leading to substantial improvements in cholelithiasis detection accuracy. Furthermore, the model exhibits robustness to variations in image quality and artifacts commonly encountered during ultrasound imaging. However, the proposed method fails to attain a high recall value within fifty epochs. We aim to address this issue in the future. The results of the proposed method are compared with those of RT-DETR and the latest versions of YOLO as benchmark models to show the efficacy of the proposed method. While RT-DETR offers faster detection, its performance degrades due to its poor localization capability in ultrasound images. The assessment of results by experienced radiologists proves the reliability of the proposed method for integration into real-world clinical settings.

## 6 Future outlook

As detection transformers mature and their applications to medical image analysis expand, several promising directions emerge for advancing the field beyond the current state of the art. This research can be extended to detect cholelithiasis through handheld devices like mobile phones and tablets by reducing storage requirements and computational overheads. In addition, the work can be extended to measure the size of gallstones to predict the severity of cholelithiasis. Furthermore, the proposed DETR model can be fine-tuned to detect other abnormalities from ultrasound images. A further frontier we see in integrating multimodal context-aware attention mechanisms, capable of fusing temporal sequences, textual annotations (e.g., radiology reports, pathology reports, [54]), and imaging metadata [55], may enhance diagnostic robustness under uncertain or noisy input conditions. This would enable context-sensitive object queries tailored to the clinical narrative

Coupling detection transformers with explainable post-hoc models such as attention saliency overlays and concept bottleneck pathways would further enhance human-in-the-loop workflows [56]. These future directions suggest a convergence toward hybrid intelligent diagnostic systems that are not only accurate and efficient but also transparent, adaptable, and clinically integrable, which we see as a hallmark of trustworthy computational science.

## CRediT authorship contribution statement

**Dheeraj Kumar:** Writing – original draft, Visualization, Software, Investigation. **Mayuri A. Mehta:** Writing – original draft, Supervision, Project administration, Methodology, Investigation, Conceptualization. **Heimo Müller:** Writing – review & editing, Validation. **Andreas Holzinger:** Writing – review & editing, Supervision, Investigation, Funding acquisition.

## Ethical approval

The method adopted in the data acquisition is as per the ethical guidelines of PGIMER, Chandigarh (Approval date: 14th July 2022), SIDS Hospital and Research Centre, Surat (Approval date: 18th January 2021), and Parul Sevashram Hospital, Vadodara (Approval date: 4th February 2020) respectively.

## Consent to Participate

This research is conducted in accordance with the ethical guidelines of life science and medical research involving human subjects. Furthermore, all experiments carried out in this research are in accordance with relevant guidelines and regulations. This research uses a collection of ultrasound images anonymously without disclosing patient information. Thus, informed consent is not required from each patient individually. Additionally, the need to obtain informed consent is waived by Parul University Institutional Ethics Committee for Human Research.

## Consent to Publish

All authors agreed with the content, and all gave explicit consent to submit. The work is original work and does not affect any copyright issues.

## Funding

Andreas Holzinger appreciates funding of the 10.13039/501100002428Austrian Science Fund, Project: https://doi.org/10.55776/P32554. For open access purposes, the authors have applied a CC BY public copyright license.

## Declaration of Competing Interest

The authors declare that they have no known competing financial interests or personal relationships that could have appeared to influence the work reported in this paper.

## Data Availability

The dataset used in this research is not available publicly due to institutional constraints associated with the contributing hospitals. The Gallbladder Cancer Ultrasound (GBCU) dataset used in this research can be obtained by submitting a request at https://gbc-iitd.github.io/data/gbcu. The dataset collected from SIDS Hospital and Research Centre, Surat, and Parul Sevashram Hospital, Vadodara can be provided on request to the authors of this paper.

## References

[1] Dai F., Cai Y., Yang S., Zhang J., Dai Y. (2025). Global burden of gallbladder and biliary diseases (1990–2021) with healthcare workforce analysis and projections to 2035. BMC Gastroenterol.

[2] Bashir S., Qamar U., Khan F.H., Naseem L. (2016). HMV: a medical decision support framework using multi-layer classifiers for disease prediction. J Comput Sci.

[3] Lian J., Ma Y., Ma Y., Shi B., Liu J., Yang Z., Guo Y. (2017). Automatic gallbladder and gallstone regions segmentation in ultrasound image. Int J Comput Assist Radiol Surg.

[4] Wang X., Yu W., Jiang G., Li H., Li S., Xie L., Bai X., Cui P., Chen Q., Lou Y., Zou L., Li S., Zhou Z., Zhang C., Sun P., Mao M. (2024). Global epidemiology of gallstones in the 21st century: a systematic review and Meta-Analysis. Clin Gastroenterol Hepatol.

[5] Beckingham I.J. (2020). Gallstones. Surg (U Kingd.

[6] Ahmed A.S., Ahmed S.S., Mohamed S., Salman N.E., Humidan A.A.M., Ibrahim R.F., Salim R.S., Mohamed Elamir A.A., Hakim E.M. (2024 Aug 8). Advancements in cholelithiasis diagnosis: a systematic review of machine learning applications in imaging analysis. Cureus.

[7] Yu C.J., Yeh H.J., Chang C.C., Tang J.H., Kao W.Y., Chen W.C., Huang Y.J., Li C.H., Chang W.H., Lin Y.T., Sufriyana H., Su E.C. (2021 Nov). Lightweight deep neural networks for cholelithiasis and cholecystitis detection by point-of-care ultrasound. Comput Methods Prog Biomed.

[8] Basu S., Gupta M., Rana P., Gupta P., Arora C. (2022). 2022 IEEE/CVF Conference on Computer Vision and Pattern Recognition (CVPR).

[9] Basu S., Gupta M., Rana P., Gupta P., Arora C. (2023). RadFormer: transformers with global–local attention for interpretable and accurate gallbladder cancer detection. Med Image Anal.

[10] Sorantin E., Grasser M.G., Hemmelmayr A., Tschauner S., Hrzic F., Weiss V., Lacekova J., Holzinger A. (2021). The augmented radiologist: artificial intelligence in the practice of radiology. Pediatr Radiol.

[11] Kumar D., Mehta M.A., Kotecha K. (2025). Computer-aided cholelithiasis diagnosis using explainable convolutional neural network. Sci Rep.

[12] Muneeswaran V., Pallikonda Rajasekaran M. (2018). Advances in Intelligent Systems and Computing.

[13] Di Ciaula A., Portincasa P. (2018). Recent advances in understanding and managing cholesterol gallstones. F1000Research.

[14] Akkus Z., Cai J., Boonrod A., Zeinoddini A., Weston A.D., Philbrick K.A., Erickson B.J. (2019). A survey of Deep-Learning applications in ultrasound: artificial Intelligence–Powered ultrasound for improving clinical workflow. J Am Coll Radiol.

[15] Jang S.I., Kim Y.J., Kim E.J., Kang H., Shon S.J., Seol Y.J., Lee D.K., Kim K.G., Cho J.H. (2021 Dec). Diagnostic performance of endoscopic ultrasound-artificial intelligence using deep learning analysis of gallbladder polypoid lesions. J Gastroenterol Hepatol.

[16] Pang S., Ding T., Qiao S., Meng F., Wang S., Li P., Wang X. (2019). A novel YOLOv3-arch model for identifying cholelithiasis and classifying gallstones on CT images. PLoS ONE.

[17] Pang S., Wang S., Rodríguez-Patón A., Li P., Wang X. (2019). An artificial intelligent diagnostic system on mobile android terminals for cholelithiasis by lightweight convolutional neural network. PLOS ONE.

[18] Obaid A.M., Turki A., Bellaaj H., Ksantini M., AlTaee A., Alaerjan A. (2023). Detection of gallbladder disease types using deep learning: an informative medical method. Diagnostics.

[19] Terven J., Cordova-Esparza D. A Comprehensive Review of YOLO: From YOLOv1 and Beyond. arXiv e-prints. 2023 Apr; arXiv:2304.00501.

[20] Sudha S., Jayanthi K.B., Rajasekaran C., Sunder T. Segmentation of RoI in Medical Images using CNN- A Comparative Study. IEEE Region 10 Annual International Conference, Proceedings/TENCON. 2019; 2019-Octob:767–71.

[21] Chen Q., Zhang Y., Li S., Chen S., Lin X., Li C., Asakawa T. (2019). Mechanisms underlying the prevention and treatment of cholelithiasis using traditional Chinese Medicine. EvidBased Complement Altern Med.

[22] Dosovitskiy A., Beyer L., Kolesnikov A., Weissenborn D., Zhai X., Unterthiner T., Dehghani M., Minderer M., Heigold G., Gelly S., Uszkoreit J., Houlsby N. An Image is Worth 16x16 Words: Transformers for Image Recognition at Scale. CoRR. 2020; abs/2010.1:1–22. 〈https://arxiv.org/abs/2010.11929〉.

[23] Carion N., Massa F., Synnaeve G., Usunier N., Kirillov A., Zagoruyko S. End-to-End Object Detection with Transformers. CoRR. 2020; abs/2005.12872. 〈https://arxiv.org/abs/2005.12872〉.

[24] Zhu X., Su W., Lu L., Li B., Wang X., Dai J. (2021). Deform DETR Deform Transform EndtoEnd Object Detect.

[25] Touvron H., Cord M., Douze M., Massa F., Sablayrolles A., Jégou H. (2020). Training data-efficient image transformers & distillation through attention. CoRR.

[26] Liu Z., Lin Y., Cao Y., Hu H., Wei Y., Zhang Z., Lin S., Guo B. (2021). 2021 IEEE/CVF International Conference on Computer Vision (ICCV).

[27] Zhao Yian, Lv Wenyu, Xu Shangliang, Wei Jinman, Wang Guanzhong, Dang Qingqing, Liu Yi, Che Jie (2023). DETRs beat YOLOs on Real-time object detection. Proc IEEE/CVF Conf Comput Vis Pattern Recognit.

[28] Liu Z., Lv Q., Yang Z., Li Y., Lee C.H., Shen L. (2023). Recent progress in transformer-based medical image analysis. Comput Biol Med.

[29] Kim J.W., Khan A.U., Banerjee I. (2025). Systematic review of hybrid vision transformer architectures for radiological image analysis. J Imaging Inform Med.

[30] Aburass S., Dorgham O., Al Shaqsi J., Abu Rumman M., Al-Kadi O. (2025). Vision transformers in medical imaging: a comprehensive review of advancements and applications across multiple diseases. J Imaging Inform Med.

[31] Slimi T., Baoues E.B., Khalifa A.B. (2025). Innovative ultrasound image denoising using channel attention and variational autoencoders. Crit Rev Biomed Eng.

[32] Kumar A., Yadav S.P., Kumar A. (2025). An improved feature extraction algorithm for robust swin transformer model in high-dimensional medical image analysis. Comput Biol Med.

[33] Chen T., Tu S., Wang H., Liu X., Li F., Jin W., Liang X., Zhang X., Wang J. (2020). Computer-aided diagnosis of gallbladder polyps based on high resolution ultrasonography. Comput Methods Prog Biomed.

[34] Jeong Y., Kim J.H., Chae H.D., Park S.J., Bae J.S., Joo I., Han J.K. (2020). Deep learning-based decision support system for the diagnosis of neoplastic gallbladder polyps on ultrasonography: preliminary results. Sci Rep.

[35] Chi J., Walia E., Babyn P., Wang J., Groot G., Eramian M. (2017). Thyroid nodule classification in ultrasound images by Fine-Tuning deep convolutional neural network. J Digit Imaging.

[36] Abdolali F., Kapur J., Jaremko J.L., Noga M., Hareendranathan A.R., Punithakumar K. (2020). Automated thyroid nodule detection from ultrasound imaging using deep convolutional neural networks. Comput Biol Med.

[37] Setiawan W., Damayanti F. (2020). Layers modification of convolutional neural network for pneumonia detection. J Phys Conf Ser.

[38] Alzubaidi L., Fadhel M.A., Al-Shamma O., Zhang J., Santamaría J., Duan Y., R. Oleiwi S. (2020). Towards a better understanding of transfer learning for medical imaging: a case study. Appl Sci.

[39] Kumar D., Mehta M.A., Joshi V.C. (2024). Empirical evaluation of filter pruning methods for acceleration of convolutional neural network. Multimed Tools Appl.

[40] Lin T.Y., Maire M., Belongie S.J., Bourdev L.D., Girshick R.B., Hays J., Perona P., Ramanan D., Dollár P., Zitnick C.L. (2014). Microsoft COCO: common objects in context. CoRR.

[41] Betancourt Tarifa A.S., Marrocco C., Molinara M., Tortorella F., Bria A. (2023). Transformer-based mass detection in digital mammograms. J Ambient Intell Humaniz Comput.

[42] Leng B., Wang C., Leng M., Ge M., Dong W. (2023). Deep learning detection network for peripheral blood leukocytes based on improved detection transformer. Biomed Signal Process Control.

[43] Obeid A., Mahbub T., Javed S., Dias J., Werghi N., Qin W., Zaki N., Zhang F., Wu J., Yang F. (2022). Computational Mathematics Modeling in Cancer Analysis.

[44] Zhang Y., Dong H., Konz N., Gu H., Mazurowski M.A., Ali S., van der Sommen F., Papież B.W., van Eijnatten M., Jin Y., Kolenbrander I. (2022). Cancer Prevention Through Early Detection.

[45] Gheflati B., Rivaz H. (2022). 2022 44th Annual International Conference of the IEEE Engineering in Medicine & Biology Society (EMBC).

[46] Li L., Wu Z., Liu J., Wang L., Jin Y., Jiang P., Feng J., Wu M. (2022). 2022 IEEE International Conference on Bioinformatics and Biomedicine (BIBM).

[47] Perera S., Adhikari S., Yilmaz A. POCFormer: A Lightweight Transformer Architecture for Detection of COVID-19 using Point of Care Ultrasound. 2021.

[48] Jiang J., Lin S. COVID-19 Detection in Chest X-ray Images using Swin-Transformer and Transformer in Transformer. arXiv preprint arXiv:2110.08427. 2021. 10.48550/arXiv.2110.08427.

[49] Liu S., Zhou H., Shi X., Pan J. Transformer for Polyp Detection. arXiv preprint arXiv:2111.07918 2021. 10.48550/arXiv.2111.07918.

[50] Mathai T.S., Lee S., Elton D.C., Shen T.C., Peng Y., Lu Z., Summers R.M. Lymph Node Detection in T2 MRI with Transformers. arXiv preprint arXiv: 2111.04885. 2021. 10.48550/arXiv.2111.04885.

[51] Shen Z., Fu R., Lin C., Zheng S. (2021). 2021 7th International Conference on Computer and Communications (ICCC).

[52] Sevinc A., Ucan M., Kaya B. (2025 Apr 4). A distillation approach to Transformer-Based medical image classification with limited data. Diagnostics.

[53] Alruily M., Mahmoud A.A., Allahem H., Mostafa A.M., Shabana H., Ezz M., Vocaturo E. (2024 Jan). Enhancing breast cancer detection in ultrasound images: an innovative approach using progressive Fine-Tuning of vision transformer models. Int J Intell Syst.

[54] Kraišniković C., Harb R., Plass M., Al Zoughbi W., Holzinger A., Müller H. (2025). Fine-tuning language model embeddings to reveal domain knowledge: an explainable artificial intelligence perspective on medical decision making. Eng Appl Artif Intell.

[55] Holzinger A., Haibe-Kains B., Jurisica I. (2019). Why imaging data alone is not enough: AI-based integration of imaging, omics, and clinical data. Eur J Nucl Med Mol Imaging.

[56] Metsch J.M., Saranti A., Angerschmid A., Pfeifer B., Klemt V., Holzinger A., Hauschild A.-C. (2024). CLARUS: an interactive explainable AI platform for manual counterfactuals in graph neural networks. J Biomed Inform.

